# Health Equity Factors and Healthcare-Associated Infections in Louisiana Facilities, 2022

**DOI:** 10.1017/ash.2024.119

**Published:** 2024-09-16

**Authors:** Tamia Dixon, Grace Lee, Alexa Ramirez

**Affiliations:** Louisiana Department of Health - Office of Public Health; Louisiana Department of Health

## Abstract

**Background:** Health equity is a critical consideration in public health research, emphasizing the importance of fair and just access to healthcare resources. This study explores the impact of health equity factors on the incidence rates of Central Line-Associated Bloodstream Infections (CLABSI) and Methicillin-Resistant Staphylococcus aureus (MRSA) across diverse healthcare facilities in Louisiana. **Methods:** We conducted a comprehensive analysis utilizing 2022 data from the National Healthcare Safety Network (NHSN). Fourteen healthcare facilities were randomly selected from nine regions in Louisiana, with guidance from the 2022 NHSN external validation toolkit. Key health equity factors from Health Resources and Service Administration (HRSA) were assessed, including urbanicity, MUA/P, and HPSA_Primary Care. Risk ratios were calculated to quantify the association between these health equity factors and the incidence rates of CLABSI and MRSA. **Results:** The findings reveal intriguing insights into the relationship between health equity factors and infection rates. In urban settings, the risk of CLABSI was lower (Risk Ratio: 0.634, 95% CI: 0.2442–1.646), contrasting with a significantly higher risk of MRSA (Risk Ratio: 1.7, 95% CI: 1.119–2.582). This suggests a complex interplay between urbanicity and the specific infection types. For MUA/P, no significant impact on CLABSI rates was observed (Risk Ratio: 0.963, 95% CI: 0.4225–2.195), but an increased risk of MRSA emerged (Risk Ratio: 1.652, 95% CI: 1.029–2.652). In healthcare professional shortage areas for primary care (HPSA_Primary Care), both CLABSI (Risk Ratio: 1.37, 95% CI: 0.5854–3.204) and MRSA (Risk Ratio: 2.098, 95% CI: 1.305–3.372) exhibited elevated risks, though only MRSA risk was statistically significant. **Conclusions:** This research underscores the nuanced relationship between health equity factors and infection rates in healthcare facilities. Urban settings may contribute to a lower risk of CLABSI but a higher risk of MRSA, emphasizing the need for tailored preventive strategies. Living in medically underserved areas appears to heighten the risk of MRSA, warranting targeted interventions. Additionally, healthcare professional shortage areas for primary care demonstrate potential associations with increased risks for both CLABSI and MRSA. These findings provide valuable insights for public health practitioners, policymakers, and healthcare administrators aiming to address health disparities and enhance infection control measures in diverse healthcare settings. Further research is encouraged to unravel the multifaceted dynamics influencing infection rates and to inform targeted interventions for improved health outcomes.

